# School-Based Virtual Reality Programming for Obtaining Moderate-Intensity Exercise Among Children With Disabilities: Pre-Post Feasibility Study

**DOI:** 10.2196/65801

**Published:** 2025-04-25

**Authors:** Byron Lai, Ashley Wright, Bailey Hutchinson, Larsen Bright, Raven Young, Drew Davis, Sultan Ali Malik, James H Rimmer, Pelham High Community Engagement Group, Jason Harchuck

**Affiliations:** 1 Division of Pediatric Rehabilitation Medicine, Department of Pediatrics, University of Alabama at Birmingham, 1600 7th Avenue South, Birmingham, AL, 35233, United States, 1 2056389790 ext 8-9725; 2 CMH Kharian Medical College, National University of Medical Sciences, Rawalpindi, Pakistan; 3 Dean's Office, School of Health Professions, University of Alabama at Birmingham, Birmingham, AL, United States

**Keywords:** disability, adapted physical activity, leisure-time exercise, exercise, telehealth, tele-exercise, cerebral palsy, pediatric rehabilitation, intellectual disability, developmental disability, child, high school, exercise, mobility, mobility disability, cardio, cardiorespiratory, cardiometabolic, feasibility, virtual reality, controlled trial, *t* test, VR, exergame, mixed method

## Abstract

**Background:**

Children have busy daily schedules, making school an ideal setting for promoting health-enhancing exercise behavior. However, children with mobility disabilities have limited exercise options to improve their cardiorespiratory fitness and cardiometabolic health.

**Objective:**

This study aims to test the feasibility of implementing a virtual reality (VR) exercise program for children with mobility disabilities in a high school setting.

**Methods:**

A pre- to posttrial single-group design with a 6-week exercise intervention was conducted at a high school. The study aimed to enroll up to 12 students with a disability. Participants were given the option of exercising at home or school. The exercise prescription was three 25-minute sessions per week at a moderate intensity, using a head-mounted VR display. School exercise sessions were supervised by research staff. Home exercise sessions were performed autonomously. Several implementation metrics of feasibility were recorded, including exercise attendance, volume, adverse events or problems, and benefits related to health-related fitness (walking endurance and hand-grip strength). The study also included a qualitative evaluation of critical implementation factors and potential benefits for participants that were not included in the study measures. Outcomes were descriptively analyzed, and 2-tailed *t* tests were used as appropriate.

**Results:**

In total, 10 students enrolled in the program and 9 completed the study (mean age 17, SD 0.6 y). In total, 5 (56%) participants exercised at school, and 4 (44%) exercised at home; 1 participant dropped out prior to exercise. The mean attendance for all 9 completers was 61.1% (11/18 sessions). The mean exercise minutes per week was 35.5 (SD 22) minutes. The mean move minutes per session was 17.7 (SD 11) minutes. The mean minutes per session was 18 (SD 1.4) minutes for school exercisers and 17 (SD 18) minutes for home exercisers, indicating variable responses from home exercisers. The mean rating of perceived exertion per exercise session was 4.3 (SD 2), indicating a moderate intensity that ranged from low to hard intensity. No adverse events or problems were identified. No improvements in walking endurance or hand-grip strength were observed. School exercisers achieved a higher attendance rate (83%) than home exercisers (27%; *P*<.001) and seemingly had a 2-fold increase in the volume of exercise achieved (school: mean 279, SD 55 min; 95% CI 212‐347; home: mean 131, SD 170 min; 95% CI –140 to 401; *P*=.10). Qualitative themes relating to implementation factors and benefits to participant well-being were identified.

**Conclusions:**

This study identified factors to inform an optimal protocol for implementing a high school–based VR exercise program for children with disabilities. Study findings demonstrated that moderate exercise at school is feasible in VR, but simply providing children with VR exergaming technology at home, without coaching, will not successfully engage them in exercise.

## Introduction

Children with disabilities generally participate in low volumes of physical activity [1,2], which places them at high risk for cardiometabolic disorders (eg, cardiovascular and metabolic diseases) [3-6]. Cardiometabolic disorders among people with disabilities seem to onset during the early adult years and manifest around the mid-30s at higher prevalences than people without disabilities [6-8]. Age-related trends in disease onset are believed to be linked with gradual reductions in health-enhancing doses of physical activity and accelerated physical deconditioning that is unique to people with disabilities [5,8,9]. Reduced participation in physical activity is suggested to be primarily caused by a lack of accessible, usable, and available opportunities and support within the community [10]. Given that children who participate in physical activity are more likely to participate as adults [11,12], there is a strong need to engage children with disabilities in regular physical activity behavior as a means of health prevention.

Outside of formal rehabilitation from physical and occupational therapy, the high school setting is often the last structured opportunity for physical activity in a person’s lifetime. In the United States, physical activity opportunities in high school are mandated by federal law to be provided to all children with disabilities. Nevertheless, mandated opportunity does not necessarily mean that a child will have accessible and usable exercises that can lead to meaningful improvements in health. A systematic review has demonstrated that school-based exercise studies generally have not elicited favorable effects on cardiometabolic health among children with disabilities, but improvements can be observed in cardiorespiratory and muscular fitness and other components of health-related physical fitness [13]. These findings are also supported by more overarching reviews of disability exercise studies outside of the school setting [2]. Children with mobility disabilities who cannot walk, run, or cycle for prolonged periods have very few evidenced aerobic exercise modalities for improving their cardiometabolic health and cardiorespiratory fitness [2,14,15].

Active video gaming is one of the few evidenced aerobic exercise modalities that can improve health among children with disabilities [14,15]. Past active video gaming studies typically used console-based devices such as the Nintendo Wii or X-Box Connect. However, those devices have been discontinued by their manufacturer, and the market is dominated solely by virtual reality (VR) head-mounted displays (HMDs). HMDs emerged during the COVID-19 pandemic in 2019, with the release of the Oculus Quest (now referred to as Meta Quest). The Quest was the first all-in-one HMD that allowed a high-quality gaming experience, and it could be purchased at any major retailer at a price comparable to gaming consoles. In 2019, the first study to use a VR HMD among children with disabilities demonstrated that 2 children with spina bifida could obtain health-enhancing doses of moderate-intensity exercise at home with behavioral telehealth coaching. Since then, HMDs have been used in a variety of contexts, such as pediatric rehabilitation, pain management, and mindfulness training [16-19]. One trial is currently testing the preliminary efficacy of VR HMD exercise training on cardiometabolic health in children with cerebral palsy [20]. However, to the best of our knowledge, few studies have tested the implementation of a VR HMD exercise program for children with disabilities in high school, particularly using the latest HMD, the Meta Quest 3.

The purpose of this 1-year feasibility study was to work with a community engagement group to develop and test the implementation of a VR HMD exergaming program for children with special needs at a high school. The overall purpose of this study was to identify a replicable protocol that could be implemented within the daily operations of a high school through the following research questions:

- How much exercise can students feasibly fit within a school-based program?
- How well would students attend the program?
- How many students can be anticipated to be enrolled from a single school?
- What payment incentive would be required to satisfy enrollment targets and ensure adherence to data collection?
- What were the potential effect estimates of the program on physical fitness tests?
- How do outcomes compare between exercising in school or exercising at home by using a loaned headset by the school?
- Qualitatively, what critical factors should be considered in designing a larger trial?

## Methods

### Design

This feasibility study was a community-based participatory research design with 2 phases, namely, a development phase and an implementation phase. The development phase included a 4-month preparation phase, where study staff worked with a community engagement group to modify a working VR randomized controlled trial home-based protocol to a local public high school setting. The 9-month implementation phase used a “learn-as-you-go” approach with a single group pre- to posttrial design to test the implementation of the developed VR HMD school-based protocol. The study was implemented at a public high school within the United States, which had adapted education support for children with special needs.

### Community Engagement Group

The community engagement group included a case manager of the high school, a young adult with cerebral palsy (male; 19 y of age) with mild intellectual disability and mobility disability who was a student at the school, and his caregiver. The group was involved in the study, starting from the design in the development phase to the completion of the study in the implementation phase. The group met weekly with the research staff during the development phase, then biweekly during the first half of the implementation phase, and monthly thereafter. The purpose of the community engagement group was to make protocol modifications to intervention and data collection procedures of an existing clinical intervention [20] that would best fit in real-world, daily operations at the school.

### Participants

This feasibility study aimed to recruit a convenient sample of 12 participants, which is a general recommendation for exploratory studies targeting implementation process metrics [21], as opposed to group-effect comparisons that would need larger samples [22]. Eligibility criteria are listed below. Participants were recruited through word-of-mouth and flyers by the community engagement group (case manager and young adult).

Inclusion criteria for the study included (1) between 10 and 19 years of age, (2) enrolled in school special education or self-reported disability, (3) a note from a physician permitting participation in moderate-intensity exercise or cleared for sports participation through physical examination that was on file by the school, and (4) ability to communicate in English (or a caregiver).

Exclusion criteria for the study included (1) complete blindness or deafness and (2) conditions that may make participation unsafe.

### Supplies

Participants used a VR HMD for exercise (Quest 3, Meta, United States), which came with 2 hand-held controllers. Active video games were purchased through the Meta Oculus digital cloud server. The games were purchased for a standard Meta account created for the study. Games included Beat Saber, Les Mills Body Combat, Creed: Rise to Glory, Smash Drums, Power Beats VR, and Thrill of the Fight.

### Intervention Development and Protocol

During the development phase of the study, the community engagement group worked with the research team to modify a home-based VR HMD clinical trial protocol [20] to be more easily implemented in a high school setting. In summary, the original intervention prescription included 150 minutes of moderate-intensity activity per week using the Quest 3 at home, for a total of 12 weeks. The home exercise was supported by theory-driven behavioral telecoaching calls [20]. In that study, caregivers were the primary intervention agents who managed their child’s exercise schedules and study activities.

Through the 4-month development phase for this study, meetings with the community engagement group resulted in the following finalized exercise protocol to test in high school:

- Frequency: 3 times per week.
- Intensity: moderate intensity exercise as indicated by a rating of perceived exertion of 5 to 7 on the Borg 0‐10 scale.
- Time: as many minutes of exercise as possible that could be fit within students’ school schedules (eg, either within their physical education period or a study hall or gap period).
- Type: VR exercise at home or in the school setting, at the choice of the caregiver and participant. At enrollment, caregivers were asked whether they preferred the child to perform the exercise at school or home with a borrowed headset. Home-based participants were not given more attention than those who exercised at school. For example, no behavioral coaching calls were given to home participants as is done in the protocol from which this study was modified [20].

### Intervention Procedures

Participants and their caregivers who were interested in the program were referred to study staff by the community engagement case manager. Participants were contacted by research staff and screened through phone calls. Eligible participants were directed to a web-based electronic data capture platform (REDCap [Research Electronic Data Capture]; Vanderbilt University) to provide informed consent and assent, participant demographics, and complete the physical activity survey. Next, they were scheduled for an on-site visit at the school to complete the physical fitness tests. Next, study staff scheduled the exercise sessions within a gap period or physical education period for participants who chose to exercise at school. Exercise sessions were performed at the school for 6 weeks, and all sessions were supervised by at least 1 research team member. For participants who chose to exercise at home, they were provided with a Quest 3 with the preinstalled games in a convenient carrying case. They were provided with the same exercise prescription as those who chose to exercise in the school. Home-based participants were not provided with additional attention or behavioral coaching. Quest 3 headsets were returned to the school once the home-based participant completed the 6-week program.

### Measures

#### Participant Characteristics

Age, sex, height, weight, and ethnicity were descriptively reported for the enrolled participants. The use of a mobility device and wheelchair was recorded. As a gauge for how physically active participants were upon joining the study, physical activity level was measured using the National Institutes of Health Patient-Reported Outcomes Measurement Information System Pediatric Physical Activity Short Form 8-item survey [23]. The survey results in a total T-score. T-scores range from 20 to 80, with a mean of 50 (SD 10). A higher T-score indicates high levels of participation in physical activity. Scores that were 0.5‐1 SD below the mean indicate “mild” difference or impairment; scores 1.0‐2.0 SD below the mean indicate “moderate” impairment; and scores ≥2.0 SD below the mean indicate “severe” impairment.

#### Research Question 1: Exercise Output

Exercise volume was measured weekly by 2 variables, namely, mean “move” minutes and rating of perceived exertion. Move minutes were automatically recorded by the Quest 3. The Quest 3 includes a built-in fitness tracker app, referred to as Meta Quest Move. The app tracks calories and move minutes, and these data are stored in cloud-based Meta servers through end-to-end encryption. Meta does not provide specifics on how these data are calculated, but the calculations are reported by Meta to be estimated through an algorithm that includes basal metabolic rate along with the headset, controller, and hand movements. At school, the maximum possible number of active minutes that a participant could exercise each week was 75 minutes because of designated 3-hour class periods. Home-based exercise participants were asked to match the school prescription (3 sessions per week for a total of 75 min). Mean move minutes were averaged each week across the 6-week program. To provide a measure of the intensity of the exercise, participants were asked immediately after exercise to provide a single rating of perceived exertion score that summarized each session, using the Borg 0‐10 scale [24]. In summary, a score of 0 represents “nothing at all,” a score of 3 is indicative of moderate intensity, a score of 5 indicates “strong” exercise, a score of 7 indicates “very strong,” and 10 indicates “extremely strong.” The mean calories were also used as an indicator of exercise output. Adverse events or problems were reported and documented in accordance with the policies established by the university.

#### Research Question 2: Student Attendance

Student attendance was expressed as a percentage value, measured by the number of exercise sessions attended, divided by the total sessions prescribed. The exercise prescription was a total of 18 sessions, broken up into 3 sessions per week for 6 weeks.

#### Research Questions 3 and 4: Student Enrollment and Payment Incentives

Student enrollment was measured by the number of students enrolled per school semester. The program lasted a total of 2 school semesters and was completed prior to summer break. Payment incentives were planned to be increased if the target sample size of 12 was not met from the first school semester. Payment incentives started at US $50 for each of the 2 data collection visits, for a total of US $100. Because of inadequate enrollment in the first semester, the payment incentive for the second semester was increased to US $100 per data collection visit, for a total of US $200.

#### Research Question 5: Potential Effect Estimates of the Program on Physical Fitness Tests

Physical fitness was measured by walking endurance and hand-grip strength. Walking endurance was measured using a 6-minute walk test (6MWT). A 6-minute push test was planned to be used for students who used wheelchairs, but this was never used. The 6MWT measures the distance walked within 6 minutes around a designated course. The course was a long unobstructed hallway that allowed the participant to freely walk for the duration of the test. When participants reached the end of the hall, they were instructed to turn and continue down the hallway. The rationale for choosing this location was that the study staff found there was limited space to set up and conduct a larger circular track during school hours, particularly without interference from other students. The 6MWT is suggested to be valid and reliable among children without disabilities [25]. However, the 6MWT has conflicting evidence regarding validity for various pediatric clinical groups [26,27]. Hand-grip strength was measured by the mean value of 3 maximal grip trials on a hand-held dynamometer (Camry Digital Hand Dynamometer, Camry, United States), while the participant was in a seated position. Grip-strength has evidence to support its use among children with intellectual disabilities [27].

#### Research Question 6: Ideal Exercise Environment

To identify the ideal method for how schools can implement the exercise program for students in a future trial, outcomes were compared between exercise settings (home vs school). Compared outcomes included enrollment, exercise volume, attendance, and fitness tests.

#### Research Question 7: Qualitative Evaluation of Factors for Future Trials

The qualitative component of this study used a mini-ethnographic design, whereby the interventionists were immersed in the culture at school with participants. At postintervention, the interventionists were interviewed (semistructured) to gain insight into feasibility issues and possible programs that should be considered in a future trial. Specific questions were: (1) What were critical issues that impeded or facilitated the implementation of the program at school? and (2) What potential health or fitness-related benefits did participants experience throughout the program (particularly, benefits that were not measured)? When considering these questions, the interventionists were asked to recall and talk through their experience with each of the on-site exercise students. Interventionists were asked to take notes throughout the study. Interviews were audio recorded and analyzed by the lead interventionist, a qualitative analyst (BL), who had conducted more than 400 interviews related to disability and health.

### Analyses

Descriptive statistics, including means, SDs, and CIs, were obtained for all study variables. Changes in means were compared primarily using descriptive comparisons with 95% CIs, with 2-tailed *t* tests as appropriate. Analyses were performed using SPSS Statistics (version 29; IBM Corp). Qualitative data were analyzed using a thematic analysis approach [19].

### Ethical Considerations

The study was approved by the University of Alabama at Birmingham Institutional Review Board for Human Use prior to enrolling participants (FWA00005960). Participants had to provide informed consent and assent documentation prior to joining the study. The principal of the high school approved and supported the study. Participants were compensated for participation in the data collection (US $100 per data collection, total incentive of US $200). All data were deidentified.

## Results

### Participant Characteristics

A total of 10 participants enrolled in the program over the 2 semesters. One participant lost interest in joining the program and dropped out prior to starting the program. A total of 9 participants completed the data collection. A total of 5 (56%) participants chose to exercise at school, and 4 (44%) participants chose to exercise at home. Characteristics for the 9 completers are provided in Table 1. The mean age of completers was 17 (SD 0.6) years (6 male and 3 female participants). The mean total Patient-Reported Outcomes Measurement Information System physical activity T-score was 41.9 (SD 7.2), indicating that physical activity levels were mild to moderately below average. Only 1 participant used a mobility device, ankle-foot orthotics. None of the participants used a wheelchair.

**Table 1.** Participant characteristics.

ID	Age (years)	Sex	Hispanic or Latino	Ethnicity	Height (inches)	Weight (lbs)	Primary disability	Physical activity (T-score)	Exercise setting
1	17	Female	No	White	56	225	Down syndrome	43.1	School
2	18	Male	No	White	60	190	Autism	28.8	School
3	17	Male	No	White	68	195	Brain tumor	48.8	School
4	16	Male	No	White	61	145	Autism or ADHD^a^	44.6	School
5	16	Female	No	White	62	105	Unknown	43.1	School
6	17	Male	No	White	68	130	Unknown	40.4	Home
7	17	Female	No	White	62	125	ADHD	48.3	Home
8	17	Male	Yes	White	68	126	ADHD	31.8	Home
9	17	Male	No	White	67	114	Cerebral palsy	48.4	Home

a ADHD: attention-deficit/hyperactivity disorder.

### Implementation Observations

At the high school, all classes within students’ schedules were at the longest, 50-minute periods, and the shortest, 25-minute periods. All students exercised during their study hall period. The community engagement group and research staff chose to structure a 25-minute exercise session within the students’ 1-hour study hall to allow the students to have 25 minutes to spend on completing academic work. Students did not have physical education built into their class schedule, because they had completed the 1-year physical education requirement prior to joining the study. The education guidelines of the state required only 1 year of physical education across the students’ 4 years of education. The exercise setting was typically conducted in an available classroom, which sometimes required desks and chairs to be moved to allow adequate space for gameplay. The school information technology staff created a secure Wi-Fi for the headsets to log in to. School Wi-Fi was generally protected to prevent inappropriate internet use. The VR headsets required Meta accounts with an active email address. Research staff created the accounts and preinstalled games onto the accounts prior to the intervention.

### Exercise Volume Results

From the 9 completers (people who completed the data collections), the total mean move minutes across the 6 weeks was 213 (SD 136), ranging from 0 to 377 minutes. The mean move minutes per week was 35.5 (SD 22) minutes. The mean move minutes per session was 17.7 (SD 11) minutes. Excluding a person from the home exercise group who attended no sessions, the mean minutes per session was 19.9 (SD 11) minutes. The mean rating of perceived exertion per exercise session was 4.3 (SD 2), indicating that most exercise sessions were performed at a moderate intensity, which ranged from low to hard intensity. The mean calories expended per attended session was 90.9 (SD 53.8), ranging from 33 to 196. No adverse events or problems (eg, seizures, injuries, or incidental behavioral issues between participants and staff) occurred throughout the study.

### Attendance and Enrollment Results

The mean attendance for all 9 completers was 61.1% (mean of 11 sessions attended out of 18 total prescribed sessions). One completer attended zero sessions but completed the pre- and postdata collections. Two participants were enrolled during the first school semester, with a payment incentive of US $50 per data collection (total incentive of US $100). The remaining 8 participants were enrolled during the second school semester after the payment incentive was increased to US $100 per data collection (total incentive of US $200). Recruitment efforts by the research team and community engagement group were consistent between semesters.

### Physical Fitness Results

Mean 6MWT distances for the 9 completers were 447 (SD 94; range: 305‐567 meters; 95% CI 375‐518) and 469 (SD 78; range: 350‐568 meters; 95% CI 408‐529) at pre- and postintervention, respectively. The mean difference in 6MWT distance from pre to post was 22 (SD 52; *t*_8_=1.3; *P*=.24; 95% CI −17.7 to 61.8), indicating a possible trend toward improvement but no statistically significant change across time. Left grip strength at pre and postintervention was 24.9 (SD 8; range: 9‐35; 95% CI 19‐32) and 24 (SD 9; range: 8‐39; 95% CI 17‐31), respectively. Right grip strength at pre and postintervention was 26.7 (SD 8; range: 12‐40 kg; 95% CI 20‐33) and 27.7 (SD 10; range: 11‐47 kg; 95% CI 20‐35), respectively. These data indicated no difference in grip strength from pre- to postintervention for both hands.

### Comparing School and Home Settings

There did not appear to be a difference in baseline levels of physical activity participation between groups (school exercisers: mean T-score 41, SD 8; n=5; home exercisers: mean T-score 42.2, SD 7; n=4). Intervention attendance was higher among those who exercised at school (83% attendance; mean sessions attended 15, SD 2; 95% CI 13‐18) compared with home exercisers (27% attendance; mean sessions attended 4.8, SD 4; 95% CI –1 to 11), and 95% CIs indicated a difference between settings of 6‐15 sessions that were statistically significant (*P*<.001; mean difference=10.7 sessions). Reasons for nonattendance were mostly due to illness and detention, in response to poor behavior at school. The mean total move minutes among the school exercisers (279, SD 55 minutes; 95% CI 212‐347) was approximately a 2-fold increase over the home exercisers (131, SD 170 min; 95% CI –140 to 401). The 95% CIs indicated a difference between settings of –39 to 337 minutes in favor of the home exercisers, but this difference was not statistically significant (*P*=.10; mean difference=149 min). The mean minutes per session was 18 (SD 1.4) minutes for school exercisers and 17 (SD 18) minutes for home exercisers, indicating variable responses from home exercisers. There did not appear to be any visual or statistical changes between the home and school settings for physical fitness tests.

### Qualitative Evaluation of School Program Benefits

Given the small number of on-site school participants, only 4 themes resulted from thematic analysis. Two themes were related to program benefits, and 2 themes were related to implementation. First, gaming at school aided well-being. Students reported that an exergaming break amidst a full school schedule made them feel better. Specifically, gaming provided a mental break from schoolwork and social interaction stressors (eg, negatively perceived interactions with peers without disabilities). Second, participation in moderate-intensity exergaming increased energy levels throughout the program. For example, halfway through the program, 1 participant who was heavily engaged in the exercise was notably less exhausted when walking in between classrooms, and the participant took less rest in between exercise bouts. This benefit was observed halfway through the 6-week program. Moreover, participants noticeably increased their exercise intensity and gaming skills across the program.

Third, interventionists reported that supervision was necessary for the exercise sessions. Students sometimes require setup and management of their exercise space. For example, some students exercised in a classroom, which required assistance in moving around chairs to make sufficient space for gaming. In 1 case, a participant who was highly engaged in the exercise needed to be prevented from overexertion. The participant was demonstrating overexertion symptoms (eg, face turning red, breathing heavily, and balance becoming unstable), and this required interventionists to urge the participant to sit down and slow down on the exergaming movements. Fourth, designated school resources are critical factors for successful implementation. Space for exercise was limited within the high school. The school gymnasium was often not a suitable place for exergaming, considering that the space often had verbal and physical interference (accidental balls and objects rolling or flying into the play space) from other students. Empty classrooms were often the primary exercise scenarios. Interventionists noted that a designated space for exergaming would allow more time for exercise by reducing the time needed to set up a play space. Moreover, having a designated wireless internet connection specifically for the headsets aided implementation. The school staff assisted with creating a wireless network access log-in that was made specifically for the project. Although internet access was not necessary to play the games in the Quest 3 headset, games often required software updates that would alleviate bugs or issues in gameplay.

## Discussion

### Principal Findings

This study developed and piloted a school-modified VR active video gaming program in a high school among students with disabilities. Several key findings were identified that will inform a larger clinical trial. First, this study identified a feasible dose of exercise that can be obtained at school. Without making substantial changes to a student’s school schedule, 30 minutes in a single day is likely the most that can be inserted into a student’s schedule, specifically in their study hall period. This dose is similar to other feasibility studies attempting to insert external exercise programs into school settings [13,28,29]. Most schools in the United States require physical education, but requirements vary widely. The high school in this study required only 1 year of physical education, in accordance with requirements by the state. Considering these constraints and exercise setup (eg, equipping the headset, clearing space to play, and starting a game) and cooldown allowed students at school to achieve 18 minutes of moderate-intensity aerobic exercise each session. It is important to note that 18 minutes, done 3 times a week or even daily, is far lower than the national guidelines of 60 minutes of moderate to vigorous activity per day for children or even 150 minutes of moderate aerobic exercise per week for adults [30]. Nevertheless, 18 minutes of moderate exercise done 3 times per week may elicit health benefits among people with disabilities. A review of meta-analyses demonstrated that people with disabilities can obtain health benefits at far lower doses of exercise than those noted in adult guidelines [2].

Another notable finding was that the school was an ideal setting for both recruitment and exercise. Obtaining 9 students from a single high school is encouraging, considering that the mean sample size for randomized controlled trials of exercise among children with disabilities is approximately 30 children [14]. To foster strong recruitment, study findings suggest a minimum of a US $200 payment incentive for a total of 2 data collections. The US $200 payment incentive resulted in 77% (7/9) of participants from a single school semester, whereas a US $100 incentive resulted in only 2 enrolled participants for a semester. Moreover, students who exercised with supervision at school achieved strong attendance (83%; 15 out of 18 sessions), particularly when compared with nonsupervised home-based programs (27%; 4.8 out of 18 sessions). Although supervision places a greater burden on school or research staff, the differences in attendance likely justify the burden. Study findings demonstrate that simply providing children with VR exergaming technology will not successfully engage them in exercise volumes that can likely lead to health benefits.

### Limitations

Regarding health and well-being benefits, a limitation of this study was the inclusion of physical performance tests instead of more concrete measures of health. Results on physical performance tests can vary due to mobility, intellectual, and behavioral differences among children with disabilities. Health assessments with stronger psychometric properties will be necessary to further investigate the health benefits of VR active gaming in school settings. Examples of more robust assessments could include a graded exercise test to measure peak oxygen consumption as an indicator of aerobic fitness, or the inclusion of biomarkers (eg, blood spot tests or blood draws) to measure changes in cardiometabolic health. Measures to support the qualitative findings could include surveys or biomarkers of well-being, stress, and anxiety. Interviews could have been conducted directly with students for more in-depth data. Another study limitation was the short 6-week duration of the intervention. Although 6 weeks of structured exercise may be sufficient to improve cardiorespiratory fitness, changes in cardiometabolic health are much harder to influence from exercise and generally require higher durations lasting several months with longer volumes of weekly exercise [2,13,29]. Future trials aiming to incorporate longer intervention durations in school settings should start participants in exercise as early as possible in a school semester, which typically lasts approximately 18 weeks. Another limitation was the small sample size and quasi-experimental design. Study findings warrant confirmation in larger trials that are randomized.

### Conclusions

This study identified a feasible VR program dose that could be fit within the daily activities of a public high school in the Southeast United States. Future research is needed to identify optimal exercise doses that can improve the physical and cardiometabolic health of children with disabilities at school, given the structural and logistical constraints of implementation.

## Abbreviations

6MWT: 6-minute walk test
HMD: head-mounted display
REDCap: Research Electronic Data Capture
VR: virtual reality
